# A permutation-based multiple testing method for time-course microarray experiments

**DOI:** 10.1186/1471-2105-10-336

**Published:** 2009-10-15

**Authors:** Insuk Sohn, Kouros Owzar, Stephen L George, Sujong Kim, Sin-Ho Jung

**Affiliations:** 1 Department of Biostatistics and Bioinformatics, Duke University Medical Center, Durham, North Carolina 27710, USA; 2 CALGB Statistical Center, Durham, North Carolina 27705, USA; 3 Skin Research Institute, AmorePacific R&D Center, Yongin 449-729, Republic of Korea; 4 R&D Center, Komipharm International Co, LTD, Kyounggi-do 429-450, Republic of Korea

## Abstract

**Background:**

Time-course microarray experiments are widely used to study the temporal profiles of gene expression. Storey *et al*. (2005) developed a method for analyzing time-course microarray studies that can be applied to discovering genes whose expression trajectories change over time within a single biological group, or those that follow different time trajectories among multiple groups. They estimated the expression trajectories of each gene using natural cubic splines under the null (no time-course) and alternative (time-course) hypotheses, and used a goodness of fit test statistic to quantify the discrepancy. The null distribution of the statistic was approximated through a bootstrap method. Gene expression levels in microarray data are often complicatedly correlated. An accurate type I error control adjusting for multiple testing requires the joint null distribution of test statistics for a large number of genes. For this purpose, permutation methods have been widely used because of computational ease and their intuitive interpretation.

**Results:**

In this paper, we propose a permutation-based multiple testing procedure based on the test statistic used by Storey *et al*. (2005). We also propose an efficient computation algorithm. Extensive simulations are conducted to investigate the performance of the permutation-based multiple testing procedure. The application of the proposed method is illustrated using the *Caenorhabditis elegans *dauer developmental data.

**Conclusion:**

Our method is computationally efficient and applicable for identifying genes whose expression levels are time-dependent in a single biological group and for identifying the genes for which the time-profile depends on the group in a multi-group setting.

## Background

Time-course microarray experiments are widely used to study the temporal profiles of gene expression. In these experiments, the gene expressions are measured across several time-points, enabling the investigator to study the dynamic behavior of gene expressions over time.

A number of statistical methods have been developed in recent years for identifying differentially expressed genes from time-course microarray experiments. Park *et al*. [1] proposed a permutation-based two-way ANOVA to compare temporal profiles from different experimental groups. Luna and Li [2] proposed a statistical framework based on a shape-invariant model together with a false discovery rate (FDR) procedure for identifying periodically expressed genes based on microarray time course gene expression data and a set of known periodically expressed guide genes. Storey *et al*. [3] represented gene expression trajectories using natural cubic splines and then compared the goodness of fit of the model under the null hypothesis to that under alternative hypothesis. The null distribution of these statistics was approximated through a bootstrap method. Di Camillo *et al*. [4] proposed test statistics using the maximum distance between two time trajectories or comparing the areas under two time course curves. Approximating the null distribution of the test statistics using a bootstrap method, they show that their test statistics are more powerful than Storey *et al*. [3] if the number of measurement time points is small. Hong and Li [5] introduced a functional hierarchical model for detecting temporally differentially expressed genes between two experimental conditions for cross sectional designs, where the gene expression profiles are treated as functional data and modelled by basis function expansions. Angelini *et al*. [6] modelled time-course data within a framework of a Bayesian hierarchical model and use Bayes factors for testing purposes.

Permutation resampling methods have been popularly used to derive the null distribution of high-dimensional test statistics while preserving the complicated dependence structure among genes in microarray data analysis. In this paper, we present a permutation-based multiple-testing method for time-course microarray experiments when independent subjects contribute gene expression data at different time points. While the method can be generalized to broad class of goodness-of-fit test statistics for regression curves, for illustration we use the F-test type statistic based on natural splines used by Storey *et al*. [3]. We propose computationally efficient algorithms for identifying the genes whose expression levels are time-dependent in a single biological group and for identifying the genes whose time-profile differs among different groups. For the multiple group setting, we will consider two sets of hypotheses. In the first set, any difference among the curves, including vertically shifted parallel curves, is considered to constitute a discrepancy among the groups. For the second set, only differences in the actual time-trends are considered to be of interest after removing the vertical shift. We shall refer to these as "time-course" and "time-trend" hypotheses, respectively. Note that if two separated curves can be overlapped by a vertical shift, then they have different time-courses, but the same time-trend. The test on a time-trend hypothesis will remove potential batch effects in microarray experiments.

The rest of the article is organized as follows. We first present a non-parametric test method to identify differential gene expression in a time-course microarray. We then present simulation results to evaluate the statistical properties of the proposed method. Next, we apply the proposed method to the *Caenorhabditis elegans *dauer developmental data [7]. Lastly, we give a brief discussion of the methods.

## Methods

At first, we briefly review a smoothing method to estimate a gene expression profile over time. Using the smoothing method, we discuss a non-parametric test method for identifying genes whose expression levels are time-dependent in a single biological group and for identifying the genes for which the time-profile depends on the group among multiple groups. We approximate the null joint distribution of the test statistics using a permutation method.

### Estimation of the Time-Course Profile

Suppose that subject *i*(= 1,..., *n*) contributes gene expression levels on *m *genes (*y*_*i*1_,..., *y*_*im*_) at time *t*_*i*_. For gene *j*(= 1,..., *m*), we consider a time trajectory model **E**(*y*_*ij*_|*t*) = *μ*_*j*_(*t*), where *μ*_*j*_(·) is the unknown function that is parameterized by an intercept plus a *p*-dimensional linear basis:



Here [*W*_1_(*t*),..., *W*_*p*_(*t*)] is a pre-specified *p*-dimensional basis that is common to all *m *genes, and *β*_*j *_= [*β*_0, *j*_, *β*_1, *j*_,..., *β*_*p*, *j*_]^*T *^is a (*p *+ 1)-dimensional vector of unknown parameters for gene *j*. Similar to Storey *et al*. [3], we employ a B-spline basis (see chapter IX in de Boor [8]) and place the knots at the 0,1/(p - 1), 2/(p - 1),...,(*p *- 2)/(p - 1), 1 quantiles of the observed time points.

Let



**W **denotes the design matrix based on the spline model. Then, the least square estimator of *β*_*j *_is obtained by  = (**W**^*T*^**W**)^-1^**W**^*T*^**y**_*j*_, where **y**_*j *_= (*y*_1*j*_,..., *y*_*nj*_)^*T*^.

### One Group Case

In the case of a single biological group (*K *= 1), we often want to discover genes whose expression levels are time-dependent. For gene *j*(= 1,..., *m*), we want to test the hypotheses



against



Under *H*_*j*_, the constant is estimated as . Under , we obtain the estimate , where () is estimated as described in the previous section.

For gene *j*, the sum of squares of errors (SSE) is expressed as . Let  and  denote the SSE under *H*_*j *_and , respectively. Storey *et al*. [3] employ the F-statistic



for testing *H*_*j *_against . It is noted that for the permutation-based multiple testing described below, the (*n *- *p *- 1)/*p *factor in the *F*_*j *_test statistic will have no impact on the results and as such can be omitted from the computations.

In order to generate the null distribution of the vector of test statistics (*F*_1_,..., *F*_*m*_) for the *m *genes, we randomly match the microarray of *n *subjects {(*y*_*i*1_,..., *y*_*im*_), *i *= 1,..., *n*} with their measurement times {*t*_1_,..., *t*_*n*_} at each permutation. Let (,..., *ñ*) be a permutation of (1,..., *n*). Then {(, *y*_*i*1_,..., *y*_*im*_), *i *= 1,..., *n*} is a permutation sample of the original data {(*t*_*i*_, *y*_*i*1_,..., *y*_*im*_), *i *= 1,..., *n*}.

Family-wise error rate (FWER) is defined by the probability of rejecting any null hypothesis *H*_*j *_when all *m *null hypotheses are true. A single-step multiple testing procedure controlling the FWER at *α *level can be described as follows, refer to e.g., Westfall and Young [9] and Jung *et al*. [10].

### Multiple Testing for Time Trend of One Group

1. Compute the the F-test statistics (*f*_1_,..., *f*_*m*_) from the original data.

2. From the *b*-th permutation data (*b *= 1,..., *B*), compute the F-test statistics ().

3. Single-step procedure to control the FWER

(a) From the *b*-th permutation data, calculate *u*_*b *_= max_1≤*j*≤*m *_.

(b) For gene *j*, calculate the adjusted p-value by , where *I*(·) is an indicator function.

(c) For a specified FWER level *α*, discover gene *j *if  <*α*.

False discovery rate (FDR) is another popular type I error for multiple testing adjustment that is defined by the expected value of the proportion of the number of erroneously rejected null hypotheses among the total number of rejected null hypotheses, refer to Benjamini and Hochberg [11]. A multiple testing procedure to control the FDR at *α *level can be obtained by replacing Step 3 in above algorithm with Step 3' as described below, refer to Tusher *et al*. [12] and Storey [13].

3'. Multuple testing controlling the FDR:

 (a) For gene *j*, estimate the marginal p-value by *p*_*j *_= *B*^-1 ^ ≥ *f*_*j*_).

(b) For a chosen constant *λ *∈ (0, 1), such as 0.95 [13], estimate the q-value of gene *j *by



(c) For a specified FDR level *α*, discover gene *j *(or reject *H*_*j*_) if *q*_*j *_<*α*.

The testing algorithm can be considerably simplified during permutations. First,  is invariant under permutations, and as such one does not have to re-calculate  for the permutation samples. Second, suppose that we fix the gene expression data {(*y*_*i*1_,...., *y*_*im*_), *i *= 1,..., *n*} and shuffle the measurement times *t*_1_,..., *t*_*n *_in each permutation. Let **I **denote the *n *× *n *identity matrix. Then, noting that  = {**I **- **W**(**W**^*T*^**W**)^-1^**W**^*T*^}**y**_*j*_, permutation replicates of  can be obtained by simply permuting the columns of **I **- **W**(**W**^*T*^**W**)^-1^**W**^*T*^. Thus, **I **- **W**(**W**^*T*^**W**)^-1^**W**^*T *^does not have to be re-computed for the permutation samples. Furthermore, given that *m *is considerably larger than *n*, permuting the columns of **I **- **W**(**W**^*T*^**W**)^-1^**W**^*T*^, a matrix of dimension *n *× *n*, is more efficient than permuting the rows of [**y**_1_,...,**y**_*m*_], a matrix of dimension *m *× *n*.

### *K *Group Case

In order to compare the time-course profiles of gene expression measurements among different experimental groups, we assume that a fixed number of measurement times are pre-specified commonly among the *K *groups and at least one subject is assigned to each time point from each group. Let *t*_1 _< ⋯ <*t*_*L *_denote the *L *time points chosen, and *n*_*kl *_denote the number of patients from group *k*(= 1,..., *K*) observed at time *t*_*l*_(*l *= 1,..., *L*). We use the notations *n*_*k*· _=  to denote the number of patients from group *k *and *n*_·*l *_=  to denote the number of patients at time point *l*. So,  denotes the total number of subjects in the study. The design and sample size under each condition is summarized in Table 1.

**Table 1:** Design and sample sizes for a *K *group case.

	**Time**			
		
**Group**	***t***_**1**_	**⋯**	***t***_***L***_	**Total**
1	*n*_11_	⋯	*n*_1*L*_	*n*_1·_
⋮	⋮	⋮	⋮	⋮
*K*	*n*_*K*1_	⋯	*n*_*KL*_	*n*_*K*·_

Total	*n*_·1_	⋯	*n*_·*L*_	*n*

Let (*y*_*kli*1_,..., *y*_*klim*_) denote the expression measurements for *m *genes at time *t*_*l*_(*l *= 1,..., *L*) from subject *i*(= 1,..., *n*_*kl*_) belonging to group *k*(= 1,..., *K*). The expression values are modelled as



where *μ*_*kj*_(*t*) = *β*_0, *kj *_+ .

In the *K*-group setting, we want to identify the genes with different time profiles in different groups. The hypotheses for gene *j *are specified as



against



Under , the estimator  is estimated from the group *k *data, {(*t*_*l*_, *y*_*klij*_), 1 ≤ *i *≤ *n*_*kl*_, 1 ≤ *l *≤ *L*}. Let .

Under *H*_*j *_: *μ*_1*j*_(*t*) = ⋯ = *μ*_*Kj*_(*t*)(= *μ*_*j*_(*t*)), the group-free estimator  is estimated using the pooled data, {(*t*_*l*_, *y*_*klij*_), 1 ≤ *i *≤ *n*_*kl*_, 1 ≤ *k *≤ *K*, 1 ≤ *l *≤ *L*}. Let  denote the estimator of the common time trajectory under *H*_*j*_.

For gene *j*, the SSE under *H*_*j *_is calculated as , where (*t*) is the estimate of *μ*_1*j*_(*t*) = ⋯ = *μ*_*Kj*_(*t*) from the pooled data. The SSE under  is calculated as , where (*t*) is the estimate of *μ*_*kj*_(*t*) from the group *k *data.

We reject *H*_*j *_in favor of  for a large value of the F-statistic



The null distribution of the test statistics (*F*_1_,..., *F*_*m*_) is approximated using a permutation method. A permutation sample is generated by permuting the gene expression data within each time point: the gene expression data of *n*_·*l *_subjects at time *t*_*l*_, {(*y*_*kli*1_,..., *y*_*klim*_), 1 ≤ *i *≤ *n*_*kl*_, 1 ≤ *k *≤ *K*} are randomly partitioned into *K *groups of size *n*_1*l*_,..., *n*_*Kl*_. The subjects at different time points are not permuted. For each subject, the random vector (*y*_*kli*1_,..., *y*_*klim*_) is counted as a data point, so that the *m *genes are not permuted. One permutation sample is obtained by conducting this permutation process for all *L *time points. Note that there are



different permutations. Table 2 demonstrates a permutation when *K *= 2. The proposed restricted permutation maintains the time trend in the whole population and allows heteroscedastic error models. Multiple testing to control FDR or FWER is conducted as in one group case, but by using the *K *group *F *statistics and permuting the observed expressions within each time-point. We can save computing time by utilizing the fact that the design matrices of the regression models are invariant to permutations.

**Table 2:** Illustration of a permutation for a *K *= 2 group case

**(a) Original Data**						
		**Time**				
		
		***t***_**1**_	**⋯**	***t***_*l*_	**⋯**	***t***_***L***_

Group	1	*y*_11_		*y*_*l*1_		*y*_*L*1_
		*y*_12_		*y*_*l*2_		*y*_*L*2_
		*y*_13_				

	2	*y*_14_		*y*_*l*3_		*y*_*L*3_
		*y*_15_		*y*_*l*4_		*y*_*L*4_
				*y*_*l*5_		

**(b) A permuted Sample**						
		**Time**				
		
		***t***_**1**_	**⋯**	***t***_***l***_	**⋯**	***t***_***L***_

Group	1	*y*_14_		*y*_*l*2_		*y*_*L*3_
		*y*_12_		*y*_*l*3_		*y*_*L*1_
		*y*_15_				

	2	*y*_13_		*y*_*l*1_		*y*_*L*4_
		*y*_11_		*y*_*l*4_		*y*_*L*2_
				*y*_*l*5_		

## Results

### Simulations

In this section, we investigate the performance of our method for control of the FWER and power using extensive simulations. We also apply the proposed methods to a real data set.

### Simulation Study

The three scenarios considered are based on amplitude variation, phase variation and a homoscedastic versus a heteroscedastic error model. We restrict ourselves to the single- and two-group (i.e., *K *= 2) cases.

#### Simulation Settings

We set *m *= 1, 000. Given a trend *μ*_*j*_(*t*) for gene *j*(= 1,..., *m*), expression data (*y*_1_,..., *y*_*m*_) measured at time *t *are generated by



Let *a*_1_,..., *a*_1000 _and *b*_1_,..., *b*_100 _be IID *N *(0, 1) random variables. Then, heteroscedastic error terms are generated as follows. For *l *= 1,..., 100 and *j *= 1,...,10, we generate ϵ_10(*l*-1)+*j *_= *a*_10(*l*-1)+*j *_ + *b*_*l *_.

Note that the error terms (ϵ_1_,...,ϵ_*m*_) consist of 100 independent blocks of size 10, and the error terms in block *l*(= 1,..., 100), (ϵ_10(*l*-1)+1_,...,ϵ_10*l*_), have a compound symmetry correlation structure with correlation coefficient *ρ*, which is set at 0, 0.3 or 0.6. We choose *L *= 11 measurement times *t*_*l *_= 0, 1,..., 10, and simulate 4 replications at each time point for each group.

In a single-group case, non-prognostic genes (genes under *H*_*j*_) have model *μ*_*j*_(*t*) = 0, and prognostic genes (genes under ) have *μ*_*j*_(*t*) = 4 exp(-*t*) in Simulation 1 and *μ*_*j*_(*t*) = sin(2*πt*) in Simulation 2.

**Figure 1:**
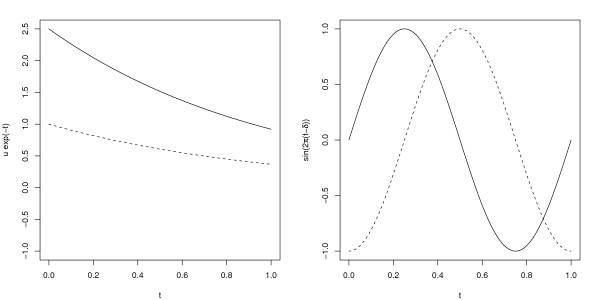
**Illustration of the amplitude (left panel) and phase variation (right panel) mean models in the two group comparisons**. The solid and dashed lines are used to distinguish the two groups.

In a two-group case (*K *= 2), we consider three different simulation models. In Simulation 1 (amplitude variation model), non-prognostic genes have equal time trends for both groups *μ*_1*j*_(*t*) = *μ*_2*j*_(*t*) = exp(-*t*), and prognostic genes have *μ*_1*j*_(*t*) = exp(-*t*) for group 1 and *μ*_2*j*_(*t*) = 2.5 exp(-*t*) for group 2, see the left panel of Figure 1. In Simulation 2 (phase variation model), non-prognostic genes have equal time trends for both groups *μ*_1*j*_(*t*) = *μ*_2*j*_(*t*) = sin(2*πt*), and prognostic genes have *μ*_1*j*_(*t*) = sin(2*πt*) for group 1 and *μ*_2*j*_(*t*) = sin(2*π*(*t *- 1/4)) for group 2, see the right panel of Figure 1.

In Simulations 1 and 2, all *m *= 1, 000 genes are non-prognostic under the global null hypothesis . Under a specific alternative hypothesis , the first *m*_1 _= 10 genes are prognostic, and the remaining *m*_0 _= 990 genes are non-prognostic.

**Figure 2:**
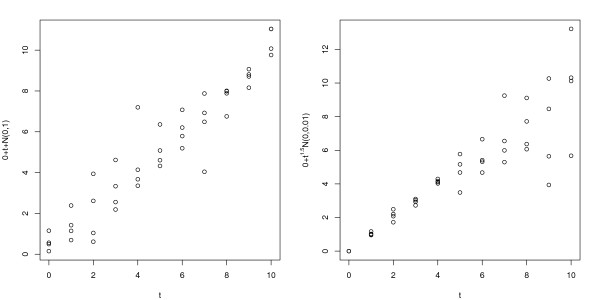
**Illustration of the homoscedastic (left panel) and heteroscedastic (right panel) error model in a two group setting**.

In Simulation 3 of a two-sample case, we consider heteroscedastic error models. Non-prognostic genes have *μ*_1_(*t*) = *μ*_2_(*t*) = *t*, and prognostic genes have *μ*_1_(*t*) = *t *and *μ*_2_(*t*) = 2.5 + *t*. For both groups (*k *= 1, 2), the first 100 genes (1 ≤ *j *≤ 100) have heteroscedastic error terms *t*^1.5 ^× ϵ_*kj*_, and the remaining 990 genes (101 ≤ *j *≤ 1, 000) have homoscedastic error terms ϵ_*kj*_. We generate (ϵ_1_,...,ϵ_*m*_) from the blocked compound symmetric multivariate normal distribution as in a homoscedastic error model. The first 5 genes with heteroscedastic error terms (1 ≤ *j *≤ 5) and the first 5 genes with homoscedastic error terms (101 ≤ *j *≤ 105) are prognostic, and all the remaining 990 genes are non-prognostic. Figure 2 displays expression levels of a non-prognostic gene under the homoscedastic error model (left panel) and under the heteroscedastic error model (right panel).

Under each setting, *N *= 1, 000 simulation samples are generated and the single-step procedure to control the FWER at 5% is applied to each sample. The null distribution of the test statistic is approximated from *B *= 1, 000 resampling (permutation or bootstrap) replications for each simulation sample. An empirical FWER under *H*_0 _(or the global power under *H*_*a*_) is obtained by the proportion of samples that reject any *H*_*j*_.

The bootstrap method by Storey *et al*. [3] generates the resampling data under null distribution as follows. We consider one group case here, but cases with *K *groups are done similarly.

1. Fit the time-course model under , and calculate *n *residuals, *e*_*ij *_= *y*_*ij *_- (*t*_*i*_).

2. Fit the time-course model under *H*_*j *_to obtain the fitted population average .

3. Generate a resampling data set under *H*_0_, {(), *i *= 1,..., *n*}, by randomly selecting the residual vectors (*e*_*i*1_,..., *e*_*im*_) among *n *subjects and adding to the vector of fitted values ().

#### Simulation Results

Simulation results are reported in Table 3 under *H*_0 _and in Table 4 under *H*_*a*_. From Table 3, we observe that both the permutation method (PERM) and the bootstrap method (BOOT) accurately control the FWER under the homoscedastic error models (Simulations 1 and 2). Under the heteroscedastic error model (Simulation 3), however, the bootstrap method is very anti-conservative, while the permutation method still control the FWER accurately. From Table 4, we observe that the two methods have almost identical global power in the homoscedastic error models. Power comparison under the heteroscedastic error model is meaningless since the bootstrap method does not control the FWER under *H*_0_.

**Table 3:** Empirical FWER level for a nominal two-sided FWER of 0.05

			**Two-group case**					
			
	**One-group case**		**Simulation 1**		**Simulation 2**		**Simulation 3**	
				
***ρ***	**PERM**	**BOOT**	**PERM**	**BOOT**	**PERM**	**BOOT**	**PERM**	**BOOT**
0	0.060	0.046	0.057	0.067	0.042	0.056	0.062	0.458
0.3	0.048	0.041	0.049	0.050	0.057	0.065	0.050	0.438
0.6	0.047	0.037	0.051	0.047	0.051	0.047	0.045	0.474

**Table 4:** Empirical global power at a two-sided FWER level of 0.05

	**One-group case**				**Two-group case**					
		
	**Simulation 1**		**Simulation 2**		**Simulation 1**		**Simulation 2**		**Simulation 3**	
					
***ρ***	**PERM**	**BOOT**	**PERM**	**BOOT**	**PERM**	**BOOT**	**PERM**	**BOOT**	**PERM**	**BOOT**
0	0.822	0.810	0.814	0.802	0.978	0.974	0.962	0.962	0.996	1.000
0.3	0.742	0.736	0.708	0.714	0.868	0.880	0.892	0.892	0.976	1.000
0.6	0.610	0.606	0.608	0.602	0.718	0.724	0.790	0.804	0.956	1.000

### Case Study

In this section, we present the results from applying our method to the analysis of the *Caenorhabditis elegans *dauer developmental data discussed by Wang and Kim [7] who use cDNA microarrays to profile gene expression differences during the transition from the dauer state to the non-dauer state (experimental group) and after feeding of starved *L*_1 _worms (control group). The cDNA microarray expressions are measured on *m *= 18,556 genes to examine the transition from dauer into normal development, where dauer animals were harvested at 0, 1.5, 2, 3, 4, 5, 6, 7, 8, 10, and 12 hours after feeding and each time point was repeated three or four times. Wang and Kim [7] perform another cDNA microarray experiment to profile gene expression at 0, 1, 2, 3, 4, 5, 6, 7, 8, 10, and 12 hours after feeding of starved *L*_1 _worms and each time point was repeated four times. This data set is available for download at . For the purpose of permutation within each measurement time, we need to unify the measurement times between groups. So, we regard the time point *t *= 1.5 in the experimental group as *t *= 1.

For this analysis, we will consider both time-course and time-trend hypotheses. A time-course hypothesis for a gene is to test any discrepancy in trajectory of its expression level over time as we have considered so far. In contrast, a time-trend hypothesis is to test any discrepancy in time trend of the gene's expression level after removing the difference in overall expression level between groups. For testing a time-trend hypothesis, the testing procedures we have discussed in the methods section can be extended by simply subtracting off group-specific means at each time point from the observed expressions first. We will contrast the results from our permutation method to those obtained by the bootstrap method suggested by Storey *et al*. [3]. Each analysis is based on *B *= 10, 000 resampling replicates and a natural spline basis as the one used in the simulation studies.

**Figure 3:**
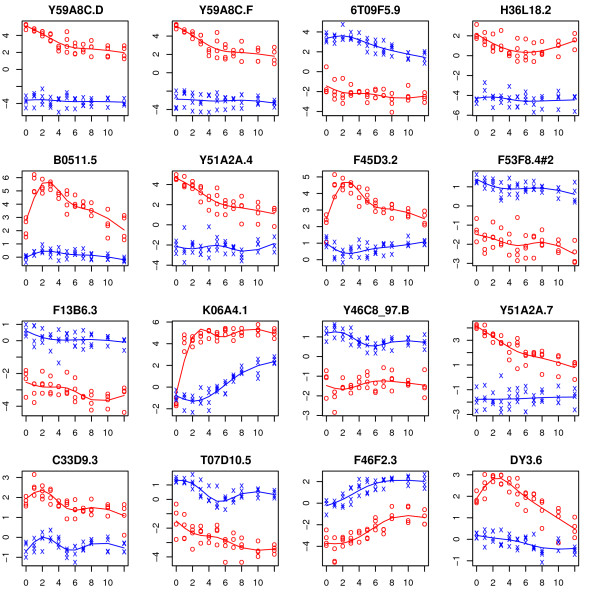
**Expression trajectories for the top sixteen genes in terms of test statistic from the Wang and Kim **[7]**data for the time-course hypothesis**. The observations from the control and experimental arms are represented by 'x' and 'o' respectively. The fitted trajectory based on a natural spline basis of dimension four is superimposed for each group (control group in blue and experimental group in red).

**Figure 4:**
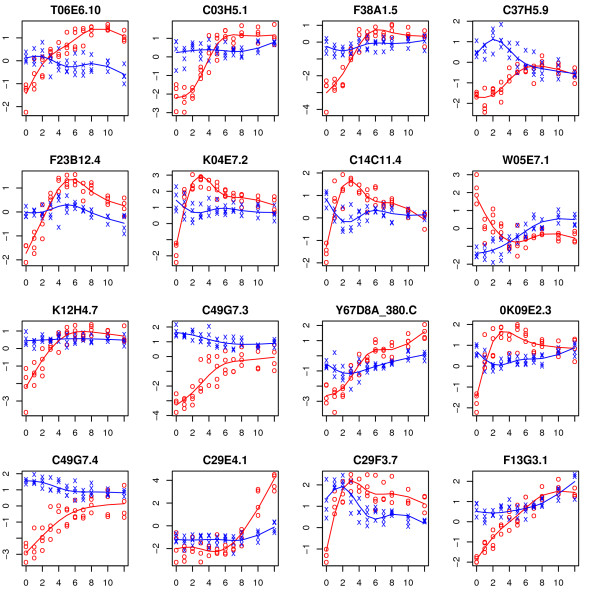
**Expression trajectories for the top sixteen genes in terms of test statistic from the Wang and Kim **[7]**data for the time-trend hypothesis**. The observations from control and experimental arms are represented by 'x' and 'o' respectively. The fitted trajectory based on a natural spline basis of dimension four is superimposed for each group (control group in blue and experimental group in red).

The top sixteen genes in terms of the realized value of the *F *statistic for testing the time-course and time-trend hypotheses are shown in Figures 3 and 4, respectively. In each case, the estimated time trajectory for each group is superimposed. For the time-course hypothesis (Figure 3), most of the top genes (e.g., *Y59A8C.D, F46F2.3*) seemingly fall into the vertical shift category while a few (e.g., *B0511.5, K06A4.1*) seemingly exhibit differing time-trends. This is perhaps not surprising as the *F *statistic tends to be largest if the curves are separated by a vertical shift. Time-trend test (Figure 4) identifies genes for which the time-trends differ between the two arms.

**Figure 5:**
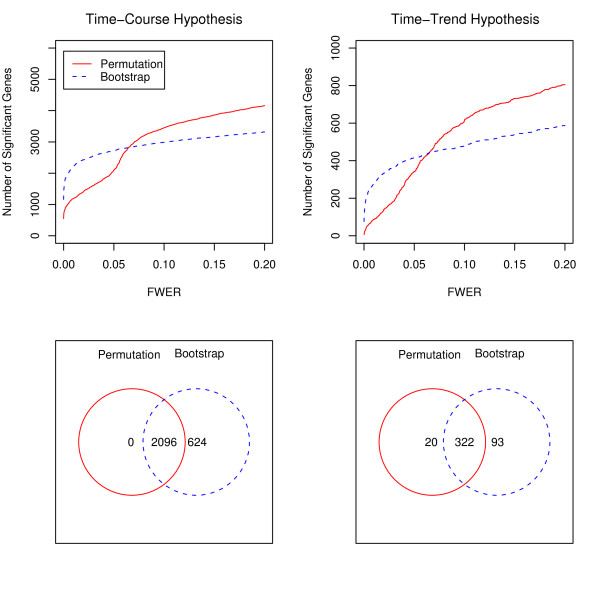
**The plots in the top row illustrate the number of significant genes for the time-course (left) and time-trend (right) hypotheses at a given FWER level (from 0 to 0.2) using permutations (solid red line) and bootstraps (dotted blue line)**. The plots in the bottom row are Venn diagrams for the number of significant genes for the time-course (left) and time-trend(right) hypotheses at 0.05 FWER level using the permutation and bootstrap methods.

Next, we will compare the result from applying our method to those obtained by employing the bootstrap approach. The number of significant genes, at a given FWER level, based on permutation and bootstrap FWER adjusted *P*-values, are shown in Figure 5 for the time-course (top-left panel) and time-trend (top-right panel) hypotheses. The permutation method tends to discover more genes for a FWER level of 0.07 or higher under both time-course and time trend hypotheses. As illustrated in Figure 5 (bottom-left panel), at the FWER level of 0.05, for the time-course hypothesis, there are 624 genes selected by the bootstrap method but not by the permutation method for the time-course hypothesis. For the time-trend hypothesis (bottom-right panel), twenty genes are identified by the permutation method but not by the bootstrap method, while 93 genes are selected by the bootstrap method but not by the permutation method. The supplementary material provides the biological properties of 13 genes (out of 20) that are identified only by the permutation method [see Additional file 1].

**Figure 6:**
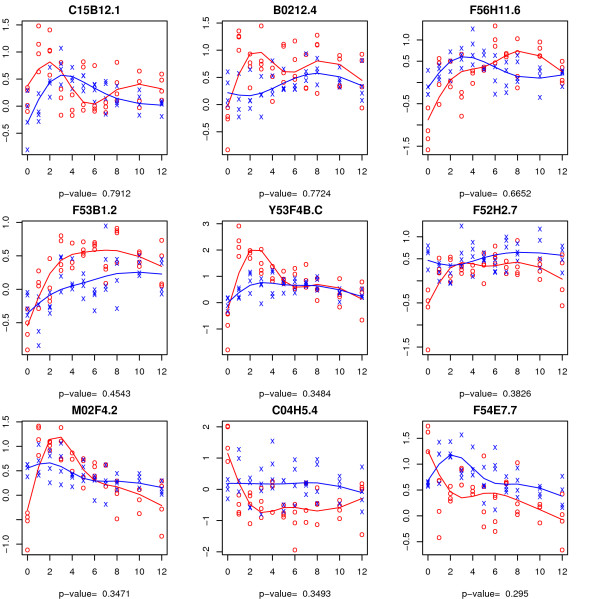
**Genes discovered by bootstrap method, but not by the permutation method, at 0.05 FWER level for the time-course hypothesis**. The observations from control and experimental arms are represented by 'x' and 'o' respectively. The fitted trajectory based on a natural spline basis of dimension four is superimposed for each group (blue for control group and red for experimental group). The adjusted *P*-value by the permutation method is provided for each gene.

**Figure 7:**
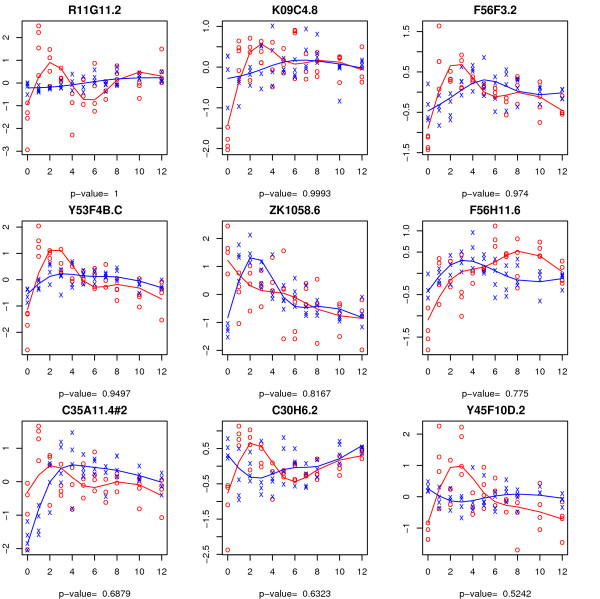
**Genes discovered by the bootstrap, but not by the permutation method, at 0.05 FWER level for the time-trend hypothesis**. The observations from the control and experimental arms are represented by 'x' and 'o' respectively. The adjusted *P*-value by the permutation method is provided for each gene.

**Figure 8:**
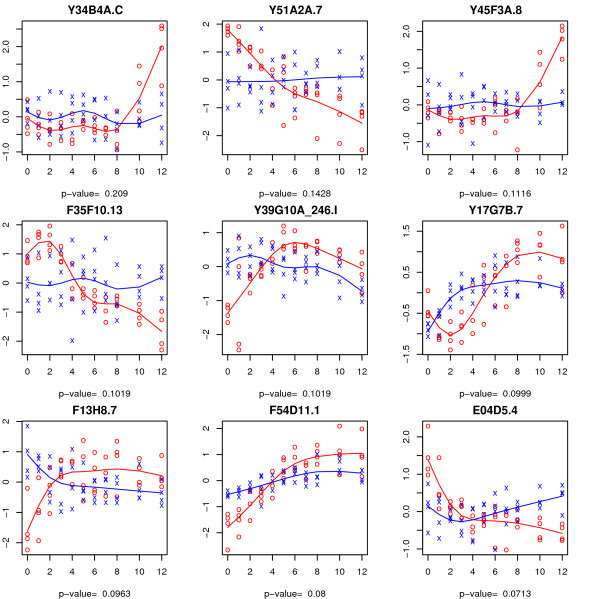
**Genes discovered by the permutation, but not by the bootstrap method, at 0.05 FWER level for the time-trend hypothesis**. The adjusted *P*-value by the bootstrap method is provided for each gene.

From each of these three sets of non-empty symmetric differences, the 9 genes with the largest difference in FWER adjusted *P*-values between the permutation and the bootstrap methods are illustrated in Figures 6 to 8. As we have illustrated in the simulation study, the bootstrap method may be severely anti-conservative if the errors are heterogeneous over time. This may explain the large set of genes that are significant according to the bootstrap method but not by the permutation method for the time-course hypothesis. The spline estimator used is not robust estimator of the regression curve in presence of outliers in which case it may give the misleading impression that the time trajectories are time dependent when in fact they are horizontal lines. Another thing to note is that, in some cases, the difference between the two time trajectories is primarily driven by their difference at the baseline, *t *= 0. It is conceivable that some of these genes would not be prognostic if the observations at baseline were to be omitted from the analysis.

## Discussion

We have considered two sets of hypotheses in the multi-group setting. For the time-course hypothesis, any difference among the groups, including parallel curves shifted vertically, would be considered interesting. For the time-trend, only cases where the time-trend is group dependent would be of interest. This method has several advantages compared to the bootstrap method suggested by Storey *et al*. [3]. First, as our simulation results have shown, the bootstrap method may not control the FWER if the error variability for each gene is heterogeneous over time. The permutation method, on the other hand, controls the FWER in the heteroscedastic case as it only requires exchangeability within time points under the null hypothesis. The bootstrap method is based on the restrictive assumption that the error model is additive and that the error terms are not only exchangeable within but also across time points.

Second, the bootstrap method requires that, in addition to matrix of observed expressions, the matrix of residuals be stored to avoid recalculating them for each bootstrap replication. Thus, compared to the permutation method, the memory requirement for the bootstrap method is about twice as large. We have illustrated our permutation method by employing the regression goodness-of-fit statistic based on natural splines used by Storey *et al*. [3]. Our method can be extended by using other regression goodness-of-fit statistics. More specifically, if one is solely interested in testing for significant genes, but not in estimating the time trajectories, then one could consider using a simple mean trace model where the time-trajectory at each point is estimated by averaging the expressions. This statistic may be more sensible if the number of time-points is small.

A referee requested that we compare the power between the *F*-statistic based on the estimated time-trajectory at each point by averaging the observations with that based on the smoothed time-trajectory proposed in this paper. For simplification, we conducted simulations in a single gene case.

For subject *i *assigned to group *k*(= 1, 2) and time *t*_*l*_(= 0, 1,...,10), the gene expression level was generated by *y*_*kli*_(*t*_*l*_) = sin[2*π*{*t*_*l *_- (*k *- 1)/4}] + ϵ_*kli*_, where ϵ_*kli *_are IID *N *(0, 1) random variables. Four subjects were assigned to each time point for each group, so that *n *= 88(= 2 × 11 × 4). We generated 10,000 simulation samples and each sample was permuted *B *= 1, 000 times. At *α *= 0.05 level, the empirical power of the statistic based on the smoothed time-trajectories was 0.9572 while that of the standard *F*-statistic is 0.8644. We observed similar comparisons under the wide range of simulation settings.

In the methods section, for the one-sample case we have proposed an efficient algorithm based on permuting columns of the projection matrix, rather than entire matrix of expressions. To evaluate the gain in efficiency empirically, we compare the two approaches for calculating FWER-adjusted *P*-values based on *m *= 10,000 genes, *n *= 100 patients and *B *= 10,000 permutations. For each approach, we replicate this simulation 10 times. The mean processing times on an AMD Opteron 8200 processor are 1,850 seconds based on permuting the matrix of expressions versus 1,764 seconds based on permuting only the columns of the projection matrix. Our approach is not only more elegant, but, as this example illustrates, may provide considerable gain in efficiency for large scale simulations such as those used in empirical power calculations where the number of simulation replicates for each design scenario and the number of markers greatly exceed 10 and 10,000 respectively.

Computer programs in R are available from .

## Conclusion

In conclusion, our permutation-based multiple testing method for time-course microarray experiments is computationally efficient and applicable for identifying the genes whose expression levels are time-dependent in a single biological group or for identifying the genes whose time-profiles are different among different groups.

## Authors' contributions

IS and KO performed statistical analysis and wrote the manuscript. SLG supported the research. SK conducted the biological interpretation of the statistical analysis results. SJ proposed the research project. All authors read and approved the final manuscript.

## Supplementary Material

Additional file 1 **Properties of 13 genes that are discovered only by the permutation method**. The data provided the biological properties of 13 genes that are discovered only by the permutation method. Click here for file
