# Supplementary material for: A permutation-based multiple testing method for time-course microarray experiments
Source: BMC Bioinformatics. 2009 Oct 15;10:336. doi: 10.1186/1471-2105-10-336 (PMC2772858; doi:10.1186/1471-2105-10-336)
Supplement: Additional file 1 — Properties of 13 genes that are discovered only by the permutation method. The data provided the biological properties of 13 genes that are discovered only by the permutation method. [file 1471-2105-10-336-S1.doc]

**Supplementary Material**

**Properties of 13 genes that are discovered only by the permutation method**

**C43C3.1 (ifp-1) gene** is induced early in the dauer exit time course, and then remains upregulated. C43C3.1 gene encodes an intermediate filament protein, a class E family member (ifp-1), which is predicted to function as a structural component of the cytoskeleton. Because many morphological and behavioral changes, such as pharyngeal pumping, movement and increased body volume occur continually during the dauer exit, cytoskeletal remodeling accompanied by cellular migration is essential. C43C3.1 gene is reported to be a positive regulator of body growth [1]. C43C3.1 gene product is shown to interact with GEI-16, which is required for ventral enclosure and elongation during embryonic and larval development, and normal rates of postembryonic growth [2]. The induction of C43C3.1 just after commitment to exit (about 1 hour) and its continuous upregulation might be important in dramatic morphological and behavioral changes over the dauer recovery process.

**Y17G7A.2 [W03C9.4 (lin-29)] gene** is also induced at the early stage of the dauer exit, and then remains induced. Y17G7A.2 [W03C9.4 (lin-29)] gene encodes a zinc finger transcription factor of the C2H2 type that regulates the heterochronic development of the vulva, egg laying system, male gonad, cuticle, and molting cycle. This gene is shown to express in terminally differentiated cells, such as uterine and vulval muscle cells, seam cells, anchor cell, vulval cells, male-specific linker cell and cells in the tail [3-7]. The manifold phenotypes, such as retardation of heterochronic alterations and growth, abnormality in locomotion, vulva development and egg laying behavior, molt defect, larval lethality, and maternal sterility are observed via RNAi suppression of Y17G7A.2 [W03C9.4 (lin-29)] gene. The induction of Y17G7A.2 [W03C9.4 (lin-29)] just after commitment to exit (about 1 hour) and its continuous upregulation might play a pivotal role in heterochronic development and growth over the dauer recovery process.

**F49E10.2 gene** is induced at the late stage of the dauer exit time course. Its biological function is unknown. However, this gene shares a region of moderate similarity with *H. sapiens* RPE65 gene product, retinal pigment epithelium-specific protein and therefore might have carotenoid oxigenase activity. The regulation of vitamin A homeostasis is very important because of the impact of vitamin A on cell differentiation, developmental processes and reproduction. The phenotype of reduced brood size was observed via RNAi suppression of F49E10.2 gene [8].

**D1054.11 gene** is a dauer-enriched gene and is downregulated gradually over dauer recovery. This gene encodes a protein of unknown function. However, the gene product is predicted to interact with proteins involved in biological processes such as reproduction and embryonic development ending in birth or egg hatching. About 50 % of its putative binding partners are integral membrane proteins regarding cellular components. About 30 % of its predicted binding partners have lipid transporter activity as molecular functions. From the function of its putative interaction partners, D1054.11 gene product might be predicted to be involved in lipid transporting process. The dauer is non-feeding and must rely on limited internal reservoirs for energy and biosynthesis. Lipid is the major source for energy and biosynthetic precursors in dauers. Therefore, genes involved in lipid transporting process must be tightly regulated during dauer recovery. Also, this gene might be involved in export and inhibition of a putative steroidal and lipophilic dauer hormone.

**Y57A10B.1 gene** shows gradual decrease in the dauer exit time course. Its biological function is also unknown. Y57A10B.1 gene has a zinc-finger domain and is predicted to be involved in mRNA turn-over and stability regulation. The phenotype of fat-content increase was observed via knockdown of Y57A10B.1 gene by RNAi [9]. Y57A10B.1 gene product might regulate the stability of mRNAs whose protein products are involved in fat storage and/or expenditure in the dauer stage.

**F13H8.7 gene** is induced early in the dauer exit time course, and then remains upregulated. This gene encodes a putative β-ureidopropionase (β-alanine synthase). This gene product has hydrolase activity acting on carbon-nitrogen (but not peptide) bonds in nitrogen compound metabolism. It is also shown to have apolipoprotein N-acyltransferase activity in lipid metabolism. The phenotype of maternal sterility was observed via RNAi of F13H8.7 gene [8]. F13H8.7 gene might be involved in nitrogen compound and/or fat metabolism during the dauer exit.

**F54D11.1(pmt-2) gene** is upregulated gradually over dauer recovery. This gene encodes an experimentally validated N-methyltransferase required for phosphocholine biosynthesis and viability. The Pmt-2 knockdown animals are unable to progress past the L1 larval stage, but can be rescued by choline in their food media [10, 11]. This result suggests Pmt-2 plays a role in nematode survival and the transition from dauer stage into next larval stage (L4).

**T27A10.3 gene** also is upregulated gradually early in dauer recovery time course. T27A10.3 gene encodes choline kinase C (ckc-1), a member of the choline/ethanolamine kinase protein family.

**Y39G10A 246.I [Y39G10AR.13 (icp-1)] gene** is induced early in the dauer exit time course, and then remains upregulated. This gene encodes putative myosin heavy chain protein, which has strong similarity to human, *D. melanogaster* and *C. elegans* myosin heavy chain protein. This gene is involved in cytokinesis, reproduction, and embryonic development ending in birth or egg hatching. The following phenotypes are observed via RNAi of Y39G10A 246.I [Y39G10AR.13 (icp-1)]; embryonic lethality, maternal sterility, abnormality in chromosomal segregation and receptor-mediated endocytosis [12-14]. During the dauer exit time course, cell division accompanied by chromosomal segregation is essential in body growth.

**Y17G7B.7(tpi-1) gene** is also upregulated gradually over dauer recovery. This gene encodes a triosephosphate isomerase. Triosephosphate isomerase (TIM) is a glycolytic enzyme that catalyzes the interconversion of dihydroxyacetone phosphate and D-glyceraldehyde-3-phosphate. The reaction is very efficient and requires neither cofactors nor metal ions. TIM is ubiquitous and conserved in function across eukaryotes, bacteria and archaea. This gene product is reported to be involved in cell redox homeostasis and determination of adult life span (Age) [15]. Knockdown of tpi-1 activity via RNAi results in increased resistance to exogenouse oxidants and shortening of natural adult lifespan, both presumably due to alterations in the redox state of cells [15, 16].

**F56F10.1 gene** is induced early in the dauer exit time course, and then remains upregulated. This gene encodes a member of the carboxypeptidase protein family. Normal worms uptake and digest food to generate energy for survival and growth. Proteases and peptidase are involved in digestion and are generally induced in the dauer exit process and remained upregulated.

**Y45F3A.8 gene** is induced at the late stage of the dauer exit time course. This gene encodes a secreted surface protein of unknown function. Knockdown of Y45F3A.8 gene via RNAi results in abnormality in embryonic and postembryonic development [17].

**E04D5.4 gene** has moderate similarities to *C. elegans* ZK673.1 ::E04D5.4::51112 and has a secreted surface protein domain.

**References**

1. Karabinos A, Schunemann J, Weber K: **Most genes encoding cytoplasmic intermediate filament (IF) proteins of the nematode Caenorhabditis elegans are required in late embryogenesis.** *Eur J Cell Biol* 2004**, 83**:457-468.

2. Li S, Armstrong CM, Bertin N, Ge H, Milstein S, Boxem M, Vidalain PO, Han JD, Chesneau A, Hao T, Goldberg DS, Li N, Martinez M, Rual JF, Lamesch P, Xu L, Tewari M, Wong SL, Zhang LV, Berriz GF, Jacotot L, Vaglio P, Reboul J, Hirozane-Kishikawa T, Li Q, Gabel HW, Elewa A, Baumgartner B, Rose DJ, Yu H, Bosak S, Sequerra R, Fraser A, Mango SE, Saxton WM, Strome S, Van Den Heuvel S, Piano F, Vandenhaute J, Sardet C, Gerstein M, Doucette-Stamm L, Gunsalus KC, Harper JW, Cusick ME, Roth FP, Hill DE, Vidal M: **A map of the interactome network of the metazoan C. elegans.** *Science* 2004, **303**:540-543.

3. Ambros V, Horvitz HR: **Heterochronic mutants of the nematode Caenorhabditis elegans.** *Science* 1984, **226**:409-416.

4. Ambros V: **A hierarchy of regulatory genes controls a larva-to-adult developmental switch in C. elegans.** *Cell* 1989, **57**:49-57.

5. Bettinger JC, Lee K, Rougvie AE: **Stage-specific accumulation of the terminal differentiation factor LIN-29 during Caenorhabditis elegans development.** *Development* 1996, **122**:2517-2527.

6. Euling S, Bettinger JC, Rougvie AE: **The LIN-29 transcription factor is required for proper morphogenesis of the Caenorhabditis elegans male tail.** *Dev Biol* 1999, **206**:142-156.

7. Abraham MC, Lu Y, Shaham S: **A morphologically conserved nonapoptotic program promotes linker cell death in Caenorhabditis elegans.** *Dev Cell* 2007, **12**:73-86.

8. Rual JF, Ceron J, Koreth J, Hao T, Nicot AS, Hirozane-Kishikawa T, Vandenhaute J, Orkin SH, Hill DE, van den Heuvel S, Vidal M: **Toward improving Caenorhabditis elegans phenome mapping with an ORFeome-based RNAi library.** *Genome Res* 2004, **14**:2162-2168.

9. Ashrafi K, Chang FY, Watts JL, Fraser AG, Kamath RS, Ahringer J, Ruvkun G: **Genome-wide RNAi analysis of Caenorhabditis elegans fat regulatory genes.** *Nature* 2003, **16**:268-272.

10. Sonnichsen B, Koski LB, Walsh A, Marschall P, Neumann B, Brehm M, Alleaume AM, Artelt J, Bettencourt P, Cassin E, Hewitson M, Holz C, Khan M, Lazik S, Martin C, Nitzsche B, Ruer M, Stamford J, Winzi M, Heinkel R, Roder M, Finell J, Hantsch H, Jones SJ, Jones M, Piano F, Gunsalus KC, Oegema K, Gonczy P, Coulson A, Hyman AA, Echeverri CJ: **Full-genome RNAi profiling of early embryogenesis in Caenorhabditis elegans.** *Nature* 2005, **434**:462-469.

11. Palavalli LH, Brendza KM, Haakenson W, Cahoon RE, McLaird M, Hicks LM, McCarter JP, Williams DJ, Hresko MC, Jez JM: **Defining the role of phosphomethylethanolamine N-methyltransferase from Caenorhabditis elegans in phosphocholine biosynthesis by biochemical and kinetic analysis.** *Biochemistry* 2006, **45**:6056-6065.

12. Simmer F, Moorman C, van der Linden AM, Kuijk E, van den Berghe PV, Kamath RS,Fraser AG, Ahringer J, Plasterk RH: **Genome-wide RNAi of C. elegans using the hypersensitive rrf-3 strain reveals novel gene functions.** *PLoS Biol* 2003, **1**:E12.

13. Romano A, Guse A, Krascenicova I, Schnabel H, Schnabel R, Glotzer M: **CSC-1: a subunit of the Aurora B kinase complex that binds to the survivin-like protein BIR-1 and the incenp-like protein ICP-1.** *J Cell Biol* 2003, **161**:229-236.

14. Fraser AG, Kamath RS, Zipperlen P, Martinez-Campos M, Sohrmann M, Ahringer J: **Functional genomic analysis of C. elegans chromosome I by systematic RNA interference.** *Nature* 2000, **408**:325-330.

15. Ralser M, Wamelink MM, Kowald A, Gerisch B, Heeren G, Struys EA, Klipp E, Jakobs C, Breitenbach M, Lehrach H, Krobitsch S: **Dynamic rerouting of the carbohydrate flux is key to counteracting oxidative stress.** *J Biol* 2007, **16**:10.

16. Gnerer JP, Kreber RA, Ganetzky B: **wasted away, a Drosophila mutation in triosephosphate isomerase, causes paralysis, neurodegeneration, and early death.** *PNAS* 2006, **41**:14987-14993.

17. Gonczy P, Echeverri C, Oegema K, Coulson A, Jones SJ, Copley RR, Duperon J, Oegema J, Brehm M, Cassin E, Hannak E, Kirkham M, Pichler S, Flohrs K, Goessen A, Leidel S, Alleaume AM, Martin C, Ozlu N, Bork P, Hyman AA: **Functional genomic analysis of cell division in C. elegans using RNAi of genes on chromosome III.** *Nature* 2000, **408**:331-336.
